# Strength Decrease, Perceived Physical Exertion and Endurance Time for Backpacking Tasks

**DOI:** 10.3390/ijerph16071296

**Published:** 2019-04-11

**Authors:** Kai Way Li, Jenn Chun Chu, Ching Chung Chen

**Affiliations:** 1 Department of Industrial Management, Chung Hua University, Hsinchu 30012, Taiwan; 2 Ph.D. Program of Technology Management, Chung Hua University, Hsin-Chu 30012, Taiwan; d1043003@gmail.com; 3 Department of Information Management, Hsing Wu University of Science & Technology, New Taipei 24452, Taiwan; 095165@mail.hwu.edu.tw

**Keywords:** manual material handling, manual operation, muscular fatigue, maximum endurance time

## Abstract

Manual material handling (MMH) tasks create a burden for workers which could result in musculoskeletal injuries. Assessments of the decrease of muscular strength and the maximum endurance time (MET) for MMH tasks are essential in studying the ergonomic risk of MMH tasks. A backpacking experiment was conducted for measuring the MET for MMH tasks. Human participants carried a load on their back and walked on a treadmill under various load, walking speed, and ramp angle conditions until they coud no longer do so. It was found that the participants were able to walk for approximately 15 min to two hours before they needed to have a pause. Their back and leg strengths declined moderately due to performing the tasks. These tasks resulted in an increase in heart rate and elevated perceived physical exertion. The rating of perceived exertion (RPE)/heart rate ratio in our backpacking tasks was 31% higher than that in the literature, implying the calibration of the RPE may be required for such tasks. A MET model incorporating the *f_MVC_back_*, body weight, walking speed, and ramp angle was established. This model may be used to determine the work/rest allowance for backpacking tasks under conditions similar to this study.

## 1. Introduction

Body pain and discomfort due to undesirable conditions such as overexertion and unnatural postures are common at work. Based on labor insurance claim data, reported injuries in the neck, shoulder, and upper extremity of workers in Taiwan increased 5.2 times in a twelve-year period (2001–2013), while lower back injuries increased 1.7 times [1]. These musculoskeletal disorders have resulted in a huge burden to both workers and the society. Due to the widespread problems of musculoskeletal disorders, the government in Taiwan stated in 2013, in the Occupational Safety & Health Actm that it is the responsibility of the employers to prevent the occurrence of these occupational injuries. Investigations of issues related to musculoskeletal injury are, therefore, urgent for maintaining a safe and healthy work environment [2].

Manual material handling (MMH) tasks are major contributors of musculoskeletal injuries in workplaces [3,4,5]. Determining the physical capability for workers is essential to the design of MMH tasks such as lifting, pushing, pulling, and carrying [6,7]. Physical capability may be determined by checking the muscular strength for a certain body segment or for a composite measure involving several body segments. Muscular strength decreases after the muscles have contracted for a period of time, resulting in the onset of muscular fatigue. 

Muscular fatigue is a common phenomenon for physical activities. It may be defined as the reduction in the ability to exert muscle force or power [8,9,10,11], failure to maintain the required or expected force [12], or failure to continue working at a given exercise intensity [13]. Muscular fatigue may be assessed by measuring the reduction of maximum voluntary contraction (MVC) after performing a forceful exertion for a period of time [14]. The reduction of the MVC, or alternatively force or strength decrease, may be quantified. A certain mathematic function may be fitted as a model describing the developing of muscular fatigue [15]. Following such an approach, muscular fatigue models have been established for various task conditions [16,17,18]. 

Ma et al. [14] suggested that a fatigue rate (*k*) for an individual or for a certain population should be incorporated when predicting the progress of muscular fatigue. The reciprocal of this parameter, or 1/*k*, was termed fatigue resistance [19]. Zhang et al. [20] assessed muscular fatigue for workers performing single arm push tasks and found that males had significantly (*p* < 0.0001) higher *k* than female participants. Muscular decrease models incorporating the concept of fatigue rate have also been employed in both one- and two-handed carrying tasks [21,22]. 

In addition to the study of muscular strength, assessment and modeling of maximum endurance time (MET) have also been beneficial to studying muscular fatigue. The MET represents the maximum time during which a static muscular load can be maintained [23]. It has been used in job assessment, especially to determine the acceptable duration of maintaining a static muscular contraction [24]. Establishment of MET models has been reported [14,19,25,26,27,28,29,30,31,32,33]. Model development involves establishing a mathematical function with the %MVC, or alternatively the relative force (*f_MVC_* = %MVC/100), as independent variable [23,34,35,36,37]. 

Most MET models in the literature were developed for static contractions. However, most tasks involving static contractions for certain body segments in industry are accompanied by dynamic or cyclic activities of other body parts. Li et al. [35] conducted a one-handed carrying study and established MET models considering body weight, walking speed, and *f_MVC_*. Their results indicated that both body weight and walking speed were important parameters in predicting the MET when walking was considered, especially when the *f_MVC_* was 0.3 or lower. 

Muscular fatigue issues for one- and two-handed carrying tasks have been discussed [21,22,35]. Backpacking is also a common manual material handling tasks, being performed, for example, by school students [38], firefighters, polices, and military personnel [39]. Theoretically, backpacking tasks involve static muscular contraction on the trunk and cyclic activities on the lower extremities. It has been hypothesized that backpacking tasks result in a decrease in muscular strength on the back and the leg due to muscular fatigue. These muscular strength decreases are believed to be associated with elevated physiological and psychophysical indices such as heart rate and subjective rating of muscular strength. The objectives of this study were to test these hypotheses. The effects of task factors such as walking speed, load carried, and ramp angle of the walk on the heart rate, decrease of muscular strength, rating of perceived exertion, and the MET for backpacking tasks were also tested. In addition, a predictive MET model incorporating walking conditions is established and discussed. 

## 2. Methods

A carrying experiment was performed in a laboratory. The temperature and relative humidity were 21.1 (±2.4) °C and 65.1 (±9.7)%, respectively. 

### 2.1. Human Participants

A total of 16 adults (10 males and 6 females) participated in the experiment. The participants were healthy without self-reported history of musculoskeletal disorders within a year. Their age, stature, and body weight (BW) were 21.7 (±2.6) years, 169.5 (±5.6) cm, and 63.0 (±11.4) kg, respectively. Signed informed consent was obtained from every participant before participating in the study. The participants were compensated 150 NTD per hour for their participation in the experiment. This study has been reviewed and approved by an external IRB (National Tsing Hua University, 10607EE061).

### 2.2. Apparatus

A treadmill which allows adjustment of inclination was used. In addition, an isometric strength measurement unit was adopted. This unit contains a platform, a chain with a handle, and a loadcell. It allows measurements of isometric back strength and isometric leg strength. This unit had been adopted in previous studies [21,22]. A Borg rating of perceived exertion (RPE) scale (6–20) was prepared to measure the perceived physical exertion of the body after a backpacking trial [40,41]. A backpack allowing to be carried with both shoulders was also prepared. The sizes of the backpack were 41 cm × 33 cm × 14 cm. The width and thickness of the shoulder straps were 7.5 cm and 0.5 cm, respectively. 

### 2.3. Experimental Conditions

#### 2.3.1. Load Carried

Carrying a load weighing 25% of their BW is common for firefighters, police officers in special missions [39], and even for school children [38]. Three load levels were tested: 0%, 12.5%, and 25% of BW. The load in the experiment was comprised with steel blocks. The load was in the backpack prepared. The participants carried the backpack on both shoulders during the trial. 

#### 2.3.2. Walking Speed

Three walking speeds were tested, including 2 km/h, 4 km/h, and 6 km/h. The walking speed was controlled by the settings on the treadmill. 

#### 2.3.3. Ramp Angle

The inclination of the treadmill can be adjusted. Two levels of treadmill inclination were tested. The first one was flat or a 0° ramp angle condition. The second was a 10° uphill condition. 

### 2.4. Procedure 

**Figure 1:**
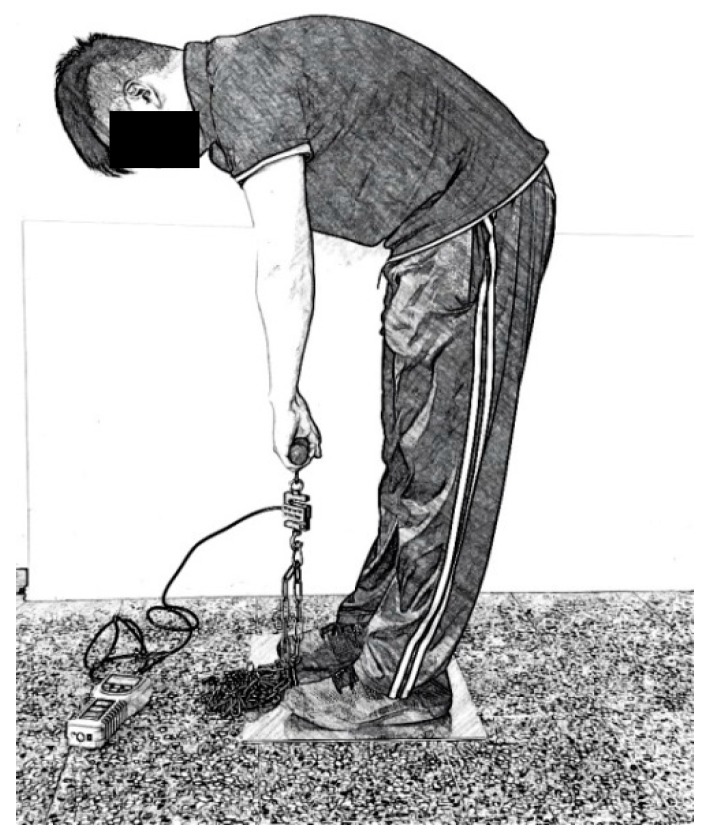
Measurement of isometric back strength.

Before the trial, the isometric back, and leg strengths were measured. For these measurements, the participant stood on a platform and pulled a handle connected to the platform upward. A loadcell was hooked between the handle and the platform to measure the force. The participant applied his/her maximum force for 4 to 6 seconds. The peak force was displayed and recorded. For isometric back strength, the participant stood and bent his/her waist to grasp the handle 38 cm above the platform and pulled upward with maximum force (see Figure 1). This force was the maximum voluntary contraction of the back muscles or *MVC_back_*. For the isometric leg strength, the participant bent his/her knee with upper body straight to grasp the handle 38 cm above the platform and pulled upward with maximum force. This force was the *MVC_leg_*. The procedure performing these strengths measurements followed those in Ayoub and Mital [42]. The participant took a break for at least five minutes after taking a strength measurement so as to avoid the effects of muscular fatigue on the next measurement.

**Figure 2:**
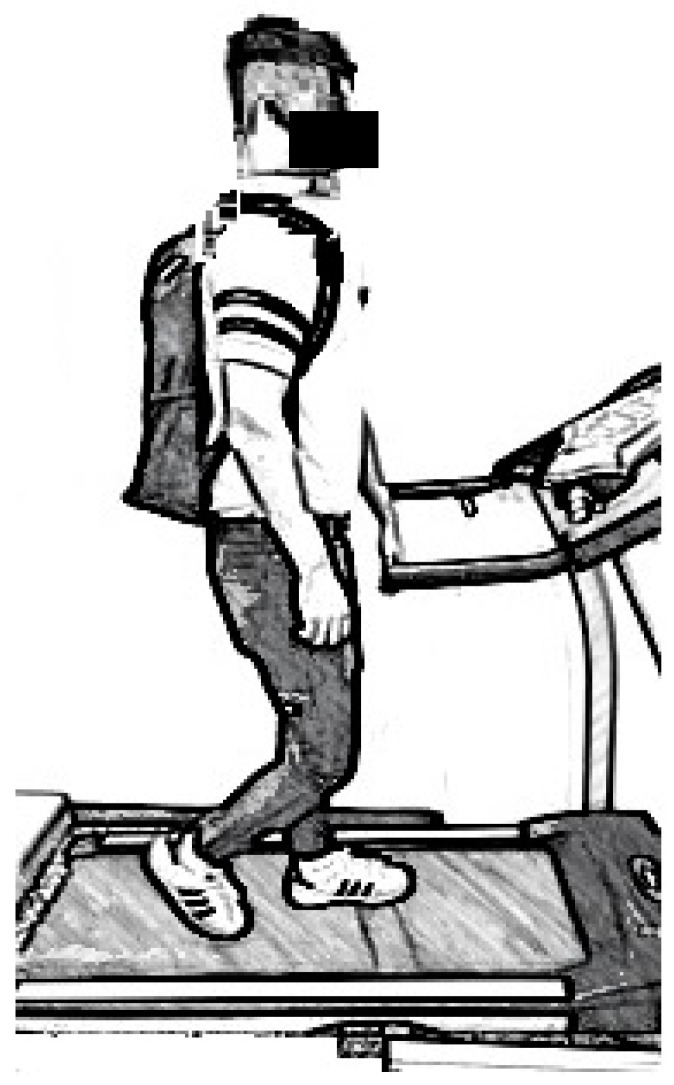
Walking with a backpack on a treadmill.

In the experiment, the participant was requested to carry a backpack with a predetermined weight and walk on the treadmill under a predetermined ramp angle and walking speed (see Figure 2). The order of the weight carried, ramp angle, and walking speed was randomly arranged in advance. The participant kept on walking until he or she could not walk any longer. Drinking and listening to music were not allowed during the trial. The heart rates of the participant before (*HR_b_*) and immediately after the walk (*HR_a_*) were recorded. The time of walking was recorded as the maximum endurance time (MET). After the walk, the participant’s RPE was immediately recorded. He or she then unloaded the backpack and was tested for his/her isometric back and leg strengths. These back and leg strengths were termed *MVC_backa_* and *MVC_lega_*, respectively. The participant joined the test only once per day to avoid the effects of fatigue. 

### 2.5. MET Modeling

Backpacking tasks involve both load carrying and walking. Load carrying on the back requires static contraction mainly on the back. Walking, on the other hand, involves dynamic contractions especially on the lower limbs. For static muscular contraction, the MET is dependent on the *f_MVC_* [14,31,36,37]. For walking, task factors which contribute to physical burdens should be incorporated. Both BW and walking speed were found to be major factors affecting energy expenditure required for walking [43,44,45]. They should be incorporated into the MET models for backpacking tasks. In addition, walking uphill requires more physical energy than walking on a level surface. Ramp angle should also be included. The MET may, then, be represented as a function of *f_MVC_*, BW, walking speed, and ramp angle:

MET = F(*f_MVC_*, *BW*, *v*, *θ*) (1)

where *v* is walking speed (km/h) and *θ* is ramp angle (degree). 

Both power and exponential functions have been proposed in MET modeling [19,23,28,36,37,46,47]. Body weight and walking speed were reported to have multiplication effects on the increment of energy expenditure for walking [45]. Equation (1) may be formulated adopting an exponential function of *f_MVC_* considering ramp angle and multiplicative effects of BW and walking speed, as in Equation (2):

(2) $$\mathrm{MET}=e^{\beta_{1}f_{MVC\_back}}\times BW^{\beta_{2}}\times {\beta_{3}}^{v}\times {\left(cos\theta \right)}^{\beta_{4}}$$

where *f_MVC_back_* is the *f_MVC_* on back muscles; *β*_1_, *β*_2_, *β*_3_, and *β*_4_ are coefficients to be determined. 

Equation (2) may be converted to a linear equation by taking natural logarithm on both sides:

(3) $$\ln\left(\mathrm{MET}\right)=\beta_{1}f_{MVC\_back}+\beta_{2}\ln\left(BW\right)+\ln\left(\beta_{3}\right)v+\beta_{4}\ln\left(cos\theta \right)$$

### 2.6. Experiment Design and Data Analysis

There was a total of 288 trials (16 participants × 2 inclined angles × 3 walking speeds × 3 load conditions). The *f_MVC_back_* was calculated by dividing the weight carried by the *MVC_back_*. The following equations were adopted to calculate the decline (%) of isometric back and leg strengths:

Back strength decline (%) = (*MVC_back_ − MVC_backa_*)/*MVC_back_* × 100% (4)

Leg strength decline (%) = (*MVC_leg_ − MVC_lega_*)/*MVC_leg_* × 100% (5)

Descriptive statistics and analyses of variance (ANOVA) were performed for the *HR_a_*, MET, RPE, and declines of both back and leg strengths. Regression analysis was performed to determine the regression coefficients in Equation (3). The statistical analyses were performed using the SAS 9.4 software (SAS Institute Inc., Cary, NC, USA). 

## 3. Results

### 3.1. Descriptive Statistics

**Figure 3:**
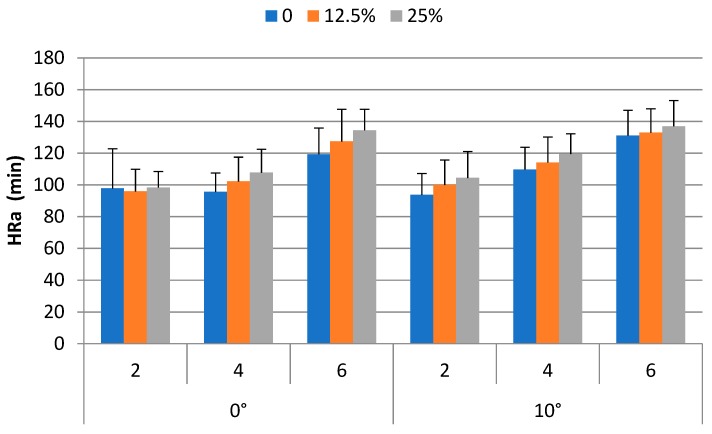
Heart rate after trial under experimental conditions.

Walking and carrying depletes our energy. Theoretically, the energy expenditure for such an activity results in increase of heart rate and decrease of muscular strength. Sixteen participants joined our backpacking experiment. Their anthropometric characteristics are shown in Table 1. Our results indicated that the heart rate of the participants increased 9.1% to 62.7% depending on the weight carried, walking speed, and ramp angle. The *HR_b_* and *HR_a_* were 85.5 (±10.3) bpm and 112.3 (±21.1) bpm, respectively. The difference between these two was statistically significant (*p* < 0.0001). This corresponds to an increase of 26.8 bpm on average. Figure 3 shows the heart rate after trial under the experimental conditions. 

**Figure 4:**
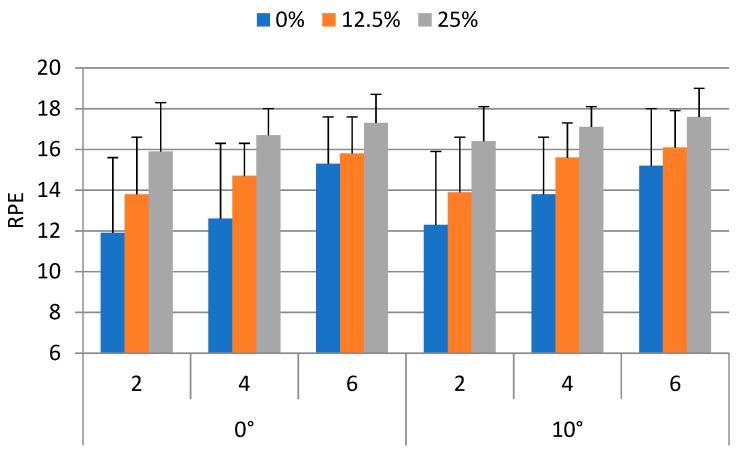
RPE under experimental conditions.

The RPE values averaged for each weight carried, walking speed, and ramp angle were between 11.9 and 17.6, with an overall average of 15.1 (±2.9). An RPE of 15 corresponds to “hard” on the RPE scale. Figure 4 shows the RPE of the participants upon completing the backpacking tasks. 

**Figure 5:**
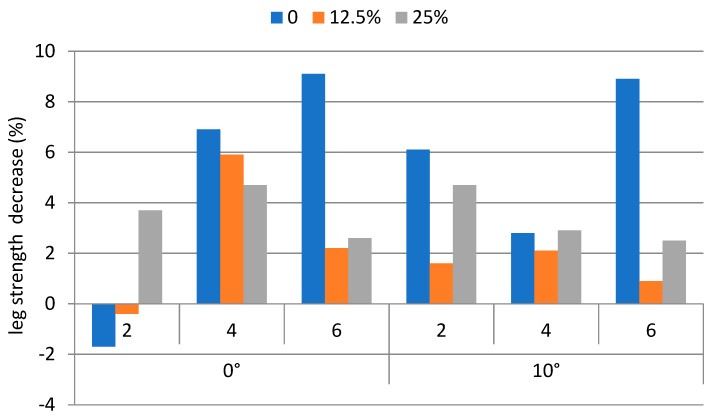
Leg strength decrease under experimental conditions.

**Figure 6:**
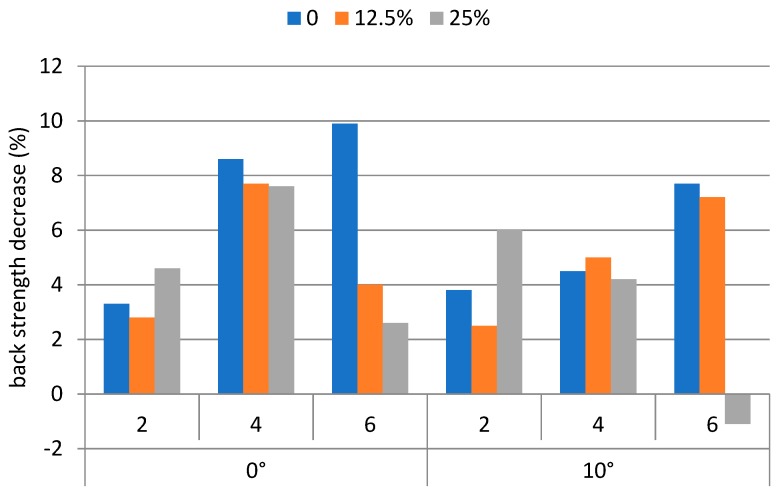
Back strength decrease under experimental conditions.

The decreases of the back strengths, averaged over each weight carried, walking speed, and ramp angle condition were between −1.1% and 9.9%. The corresponding leg strength decreases were between −1.7% and 9.1%. The strength decreases (%) on the back and the leg for performing the backpacking tasks are shown in Figure 5 and Figure 6, respectively.

**Figure 7:**
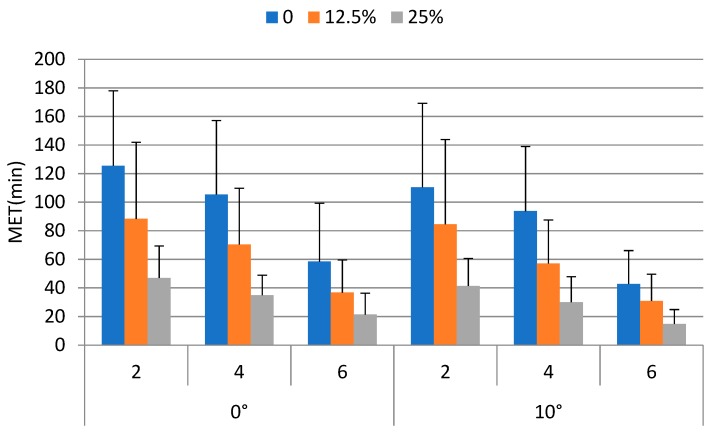
MET under experimental conditions.

Figure 7 shows the MET under experimental conditions. The MET averaged over each weight carried, walking speed, and ramp angle condition ranged from 14.8 min to 125.5 min with an overall mean (±std) of 67.3 (±48.3) min. The minimum and maximum values occurred at the least (the 0° ramp × no weight × 2 km/h) and most (10° ramp × 25% body weight × 6 km/h) strenuous conditions, respectively.

### 3.2. ANOVA Results

The ANOVA results indicated that the MET were significantly affected by the weight carried (*p* < 0.0001), ramp angle (*p* < 0.05), and walking speed (*p* < 0.0001). The effects of gender were not statistically significant. The mean MET values for the 0, 12.5% and 25% BW conditions were 89.3, 61.3, and 31.6 mins, respectively. The Duncan’s multiple range test results indicated that the MET without load carrying was significantly (*p* < 0.05) higher than those of the carrying 12.5% and 25% BW conditions. The MET of the 12.5% BW condition was significantly (*p* < 0.05) higher than that of the 25% BW conditions. The MET decreased when the walking speed increased. The mean MET values for the 2, 4, and 6 km/h walking speed were 82.8, 65.2, and 34.2 mins, respectively. The MET of the 2 km/h was significantly (*p* < 0.05) higher than those of the 4 and 6 km/h conditions. The MET of the 4 km/h was significantly (*p* < 0.05) higher than that of the 6 km/h condition. In addition, the MET for the 0° and 10° ramp conditions were 65.3 and 56.2 mins, respectively. They were significantly (*p* < 0.05) different. 

The ANOVA results indicated that *HR_a_* were significantly affected by the weight carried (*p* < 0.001), walking speed (*p* < 0.0001) and ramp angle (*p* < 0.001). The Duncan’s multiple range test results indicated that *HR_a_* of the 25%BW condition (116.9 bpm) was significantly (*p* < 0.05) higher than those of the 12.5%BW (112.1 bpm) and 0%BW (107.9 bpm) conditions. The *HR_a_* of the 12.5%BW condition and that of the 0%BW were not significantly different. The *HR_a_* for the 2 km/h (98.4 bpm) condition was significantly (*p* < 0.05) lower than those of the 4 km/h (108.1 bpm) and 6 km/h (130.3 bpm) conditions. The *HR_a_* for the 4 km/h condition was significantly (*p* < 0.05) lower than that of the 6 km/h conditions. The *HR_a_* of the 0° ramp angle condition (108.7 bpm) was significantly (*p* < 0.05) lower than that of the 10° condition (115.8 bpm). 

The ANOVA results indicated that both the decrease (%) of the back and leg strengths were insignificant to the weight carrying condition, walking speed, and the ramp angle of the treadmill. A pairwise *t*-test results indicated that back strength decrease was significantly (*p* < 0.05) higher than that of leg strength decrease. Table 2 shows the Pearson’s correlation coefficients between variables.

The ANOVA results indicated that the RPE were significantly affected by the weight carried (*p* < 0.0001) and walking speed (*p* < 0.0001). The Duncan’s multiple range test results indicated that the RPE without load carrying (13.5) was significantly (*p* < 0.05) lower than those of the 12.5% (15.0) and 25% (16.8) BW conditions. The RPE for the 12.5% BW condition was significantly (*p* < 0.05) lower than that of the 25% BW condition. The RPE when walked at 2 km/h (14.0) was significantly (*p* < 0.05) lower than those of the 4 km/h (15.1) and 6 km/h (16.2) conditions. The RPE for the 4 km/h condition was significantly (*p* < 0.05) lower than that of the 6 km/h conditions. The effects of ramp angle on RPE were not significant. 

### 3.3. MET Modeling

The following equation was obtained in the regression analysis:

(6) $$\ln\left(\mathrm{MET}\right)=-4.423f_{MVC_{back}}+1.283\ln\left(BW\right)-0.229v+11.836\ln\left(cos\theta \right)$$

This equation was statistically significant (*p* < 0.0001) with an R_adj_^2^ = 0.97. Equation (6) may be converted to Equation (7):

(7) $$\mathrm{MET}=e^{-4.423f_{MVC\_back}}\times BW^{1.283}\times 0.795^{v}\times {\left(cos\theta \right)}^{11.836}$$

A mean absolute deviation (MAD) was calculated using the following equation to compare the actual MET and predicted MET:

(8) $$\mathrm{MAD}=\frac{1}{n}\sum_{i=1}^{n}\left(actual\;MET-predicted\;MET\right)$$

The MAD of Equation (8) in estimating the MET in our backpacking tasks were 29.7 (mins).

## 4. Discussion

Muscular strength decrease has been adopted to quantify the progress of muscular fatigue [10,36,37]. Analyzing the decrease of muscular strength in a single muscle is less complicated but is of little practical use in resolving industrial problems. Assessing the muscular strength in performing a certain task is common. The task-specific assessment of muscular strength, however, involves contractions of multiple muscles. Both the isometric back and leg strengths were measured by having the participants pull a handle 38 cm above the platform, following the protocol in the literature [42]. It should be noted that the fixed handle height could lead to variations in body posture for participants with different body dimensions. It was likely that the MVC values measured using this protocol might be lower than the real MVCs of the participants, especially the taller ones. 

It was hypothesized that the strengths of the back and leg would decrease upon performing the backpacking tasks. However, both the back and leg strengths decreased only moderately (<10%). In addition, to our further surprise, the decreases of these two strengths after the trial were not affected by any of the three factors tested. An explanation of these phenomena may be that muscular strength decrease due to performing physical tasks is more likely to occur under static muscular contraction conditions. Muscular contractions on the leg were dynamic. Such dynamic contractions facilitate blood circulation, and hence slow down the onset of muscular fatigue. On the other hand, back muscle contractions were required to support the backpacking tasks. These muscles might; however, provide only partial contributions to the tasks. Many other muscles in the trunk, such as those in abdomen and shoulders, might also play a role. Unfortunately, muscular strength for those muscles could not be measured due to technical difficulties and the limitations of our apparatus. Decrease of both of the back and leg strengths were, therefore, less prominent than what we had expected. Strength decrease in the back was significantly (*p* < 0.05) higher than that on the leg, as back muscles experienced less dynamic contractions than those of the leg muscles. 

For prolonged physical activities, heart rate is one of the most appropriate physiological parameters for indicating physiological strain. The perceived physical exertion is also believed to be “the single best indicator of the degree of physical strain” [40]. It is well known that the RPE was developed based on heart rate. Even though the results indicated that walking with load carriage resulted in increased heart rate and elevated RPE ratings, the Pearson’s correlation coefficient between our RPE and *HR_a_* was only 0.3. This was much lower than those reported in the literature [41]. In addition, the RPE, averaged over all experimental conditions, at the end of the trial was 15.1. According to Borg [40], this value corresponded to a heart rate of 151 for adults aged between 30 and 50 years. Our *HR_a_*, averaged over all experimental conditions, was only 112. This might be attributed to factors other than physiological strain such as age and the type of physical exertion. Our participants were college students with a mean age of 21.7 years old. Their maximum heart rates were higher than those of the adults in Borg’s study. They might give higher RPE scores than what have been anticipated based on the literature [40]. A linear regression analysis without intercept has been performed using the RPE as the dependent variable and the *HR_a_* as the independent variable. The following equation was obtained with an R^2^ of 0.95 and *p* < 0.0001:

RPE = 0.131 × *HR_a_* (9)

The regression coefficient of 0.131 was 31% higher than the 0.1 suggested in the literature [36]. As the participants might walk up to approximately 3 hours in some trials, they could give relatively high RPE because of thirst, as the participants were not allowed to drink during the trial. In addition to age, feelings of boredom, discomfort on the shoulder due to the pressure of the strap of the pack on the local muscles, and some other reasons might also have affected the RPE reported by the participants. 

**Figure 8:**
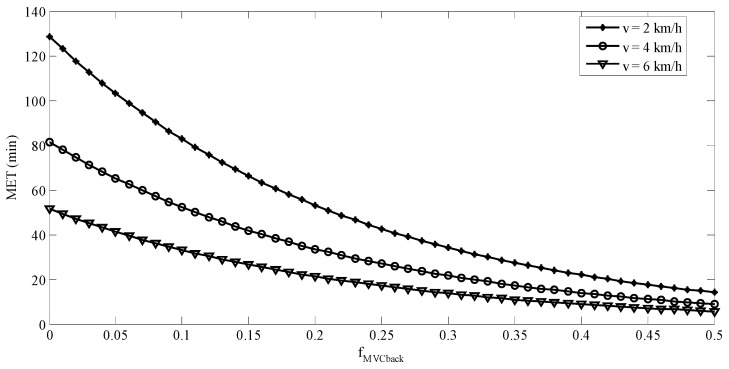
Predicted MET when θ = 0° and BW = 63 kg.

**Figure 9:**
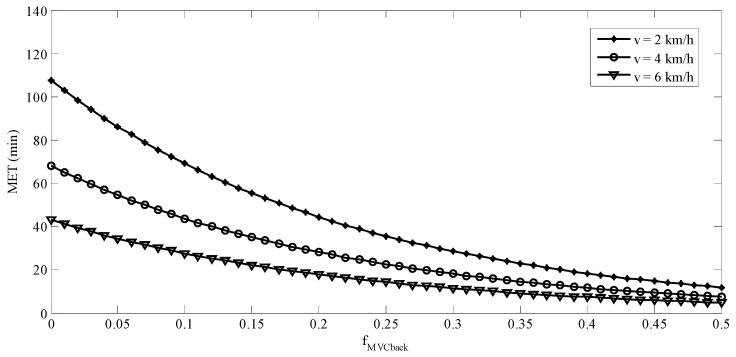
Predicted MET when θ = 10° and BW = 63 kg.

It is well known that *f_MVC_* has nonlinear effects on MET and both power and exponential functions have been adopted in the modeling of MET [36,37,46,47]. The early literature [31,32] indicated an indefinite exertion period for low *f_MVC_*. The so-called “indefinite exertion period” has been challenged for *f_MVC_* as low as 0.05 [48]. A power model of the *f_MVC_* was not considered in the current study, because it is undefined when the *f_MVC_* is equal to 0. An undefined MET function implies an indefinite MET. This is not reasonable in our backpacking tasks even there was no external load on the back. Exponential model (Equation (2)) was then fitted. In Equation (8), predictive MET was established considering *f_MVC_back_*, BW, ramp angle, and walking speed. The *f_MVC_back_*, ramp angle, and walking speed were associated with physical burdens or job demand of the tasks. Figure 8 and Figure 9 show the predicted MET using Equation (8) for ramp angles of 0° and 10°, respectively. The predicted MET values in these two figures were calculated using the average BW (63 kg) of the participants. For both ramp angles, the difference between the predicted MET values for any two walking speeds tested was becoming smaller when the *f_MVC_back_* was getting higher. The effects of walking speed on the MET were more prominent at low *f_MVC_back_* than were at a high one. This was consistent with the findings in the literature [35]. The MET model established in the current study may be used to determine the work/rest allowance for backpacking tasks similar to ours.

There were limitations in the study. First of all, the participants were not allowed to drink or listen to music. Instead of being physically exhausted, they might have given up walking because of thirst, boredom, or some other reasons. In other words, their MET values could be higher than what were recorded if they were allowed to replenish water when they were thirsty or if they were allowed to listen to their favorite music. Replenishment of water and music listening may be important factors affecting the decrease of muscular strength, RPE, and MET. Effects of such factors will be interesting topics in the future. Secondly, the experiment was conducted in the laboratory where the temperature and relative humidity were controlled at 21.1 (±2.4) °C and 65.1 (±9.7)%, respectively. It is well known that temperature and humidity do affect the physiological strain of people performing physical tasks. We believe that the MET should be lower than our prediction if the task was performed in an environment with higher temperature or humidity than ours. Thirdly, age was not considered in the modeling of MET due to the nature of our participants. Our MET model may not be applicable to people in other age groups, especially school-aged children. This is because the ranges of the body weight and walking speed of our participants are much higher than those of children. Other MET models should be established for backpacking for school-aged children in the future. Finally, the sample size of this study was not large. This was due to our budget constraint. However, a sample size of 16 was believed to be acceptable as it was larger than some of the studies in the literature [28,49]. 

## 5. Conclusions

The participants carried a backpack and walked until they could no longer do so. The endurance time for the backpacking tasks under different weight carried, walking speed, and ramp angle conditions ranged from approximately 15 min to more than two hours. These tasks resulted in an increase of heart rate and elevated perceived exertion. Our data support the hypotheses that both the MET and heart rate are affected by the weight carried, walking speed, and ramp angle. The RPE was also affected by the weight carried and walking speed significantly. The effects of ramp angle were not significant on RPE. The RPE/heart rate ratio for our backpacking tasks was 31% higher than that in the literature [40] implying the calibration of the RPE may be required for such tasks. Factors affecting the pause of backpacking tasks are very complicated. Muscular strength decreases on the back and leg might not be the predominant ones as these strengths declined only moderately at the end of the tasks. A MET model incorporating the *f_MVC_back_*, body weight, walking speed, and ramp angle was established. This model may be used to determine the work/rest allowance for backpacking tasks under conditions similar to this study.

## Figures and Tables

**Table 1:** Anthropometric characteristics of the participants.

Basic Characteristics	Male (*n* = 10)	Female (*n* = 6)
Age (years)	21.3 (1.7)	22.3 (3.4)
Stature (cm)	172.5 (5.3)	166.8 (3.8)
Body weight (kg)	67.4 (11.8)	55.7 (3.3)
Heart rate at rest (bpm)	82.5 (10.4)	90.5 (8.0)
Isometric leg strength (kgf)	62.8 (15.8)	52.7 (9.3)
Isometric back strength (kgf)	61.8 (17.2)	50.4 (7.1)

Number in parentheses are standard deviations.

**Table 2:** Pearson’s correlation coefficients.

Variable	MET	RPE	HR Increase
Weight carried	−0.49 *	0.47 *	-
Walking speed *HR_a_*	−0.41 * −0.46 *	0.31 * 0.30 *	0.62 *

* *p* < 0.0001.
